# The Effectiveness of Two Implementation Strategies for Improving Teachers’ Delivery of an Evidenced-based HIV Prevention Program

**DOI:** 10.1007/s11121-022-01335-x

**Published:** 2022-01-22

**Authors:** Bo Wang, Lynette Deveaux, Lesley Cottrell, Xiaoming Li, Richard Adderley, Barbara Dorsett, Regina Firpo-Triplett, Veronica Koci, Sharon Marshall, Nikkiah Forbes, Bonita Stanton

**Affiliations:** 1 Department of Population and Quantitative Health Sciences, University of Massachusetts Chan Medical School, 368 Plantation Street, Worcester, MA 01605 USA; 2 Office of HIV/AIDS, Ministry of Health, Shirley Street, Nassau, Bahamas; 3 Department of Pediatrics, West Virginia University, 959 Hartman Run Road, Morgantown, WV 26506 USA; 4 Department of Health Promotion, Education, and Behavior, University of South Carolina Arnold School of Public Health, 915 Greene Street, Suite 408, Columbia, SC 29208 USA; 5 Ministry of Education, Thompson Boulevard, PO Box N-3913 Nassau, Bahamas; 6 Dfusion Inc, 100 Enterprise Way, Suite D305, Scotts Valley, CA 95066 USA; 7 Department of Family Medicine and Public Health Sciences, Wayne State University School of Medicine, 6135 Woodward Ave., Detroit, MI 48202 USA; 8 Department of Pediatrics, Wayne State University School of Medicine, 400 Mack Avenue, Detroit, MI 48201 USA; 9 Founding Dean, Hackensack Meridian School of Medicine, 340 Kingsland ST, Nutley, NJ 07110 USA

**Keywords:** Implementation strategies, Evidenced-based intervention, Fidelity of implementation, HIV prevention, The Bahamas

## Abstract

**Background:**

Effective implementation strategies are needed to enhance the success of evidence-based prevention programs. The current study evaluates the effects of two implementation strategies on teachers’ implementation of an evidenced-based HIV intervention.

**Methods:**

Using our 7-item pre-implementation school screening tool, we identified teachers who were at-risk for not implementing the Focus on Youth HIV-risk reduction intervention curriculum which targets grade six through grade 8 students. After completing a two-day curriculum workshop, 81 low- and moderate-performing teachers were randomly assigned to one of four experimental conditions and were asked to teach the two-month intervention curriculum. This optimization trial examines the impact of two implementation strategies: biweekly monitoring/feedbacks (BMF) and site-based assistance/mentorship (SAM). The primary outcome is implementation fidelity defined as number of core activities taught. Linear mixed-effects model was used to examine the association of the implementation strategies with implementation fidelity.

**Results:**

BMF and SAM were significantly associated with teachers’ implementation fidelity. Teachers who received both BFM and SAM taught the greatest numbers of core activities (15 core activities on average), followed by teachers who received either BMF (6.9 activities) or SAM (7.9 activities). Teachers who did not receive BMF or SAM taught the lowest numbers (4.1 activities). Teachers’ sustained implementation of FOYC in the prior school year was related to increased implementation fidelity during the optimization trial. Teachers’ confidence in implementing five core activities, attitudes toward sex education in schools, and perceived principal support were significantly related to increased self-efficacy, which in turn was related to teachers’ fidelity of implementation before the optimization trial.

**Conclusion:**

BMF and SAM are effective in promoting teachers’ implementation of youth evidence-based interventions. Researchers and future program implementers should consider teacher training, teachers’ attitudes toward sex education, perceived principal support, and self-efficacy when attempting to maintain the effects of teacher-delivered interventions in schools.

## Introduction

### Challenges in the Implementation of Evidence-Based Interventions (EBI)

Implementation science has focused on factors and strategies that influence the adoption and implementation of EBIs in real-world settings (Proctor et al., 2015). The many challenges encountered in real-world settings result in implementation quality issues which impact program outcomes (Collins & Sapiano, 2016; Feldman et al., 2014). Compared to programs implemented in community or healthcare settings, school-based programs are especially prone to adaptations and lower implementation quality in real-world settings (Molloy et al., 2013; Forman et al., 2013).

### Factors Influencing Implementation Fidelity of EBIs in School Settings

Numerous studies have identified a range of factors that are associated with implementation fidelity: teacher training, program characteristics, teacher characteristics, and the provision of ongoing technical assistance (Mihalic et al., 2008; Renju et al., 2011; Sawyer et al., 2010). Little et al. (2013) found that comprehensive teacher training significantly increased teachers’ self-efficacy, which resulted in an increase in implementation fidelity. Teachers’ positive attitudes toward sex education predicted higher rates of school-based sex education teaching (Martínez et al., 2014). Support of school administrators, program priorities, and the values and confidence of individual teachers are primary factors which shape the implementation of school-based prevention programs (Buston et al., 2002). Factors which are inconsistent with these supporting elements undermine implementation (Forman et al., 2009). Local adaptations of interventions, variations in teacher competence, lack of available training and technical support, limited local resources for supporting the intervention, and staff absence and turnover have been identified as barriers to maintaining implementation fidelity (Botvin, 2004; Hill et al., 2007).

### Implementation Strategies Enhancing Teachers’ Implementation of EBIs in Schools

The implementation approach “*Fidelity through Informed Technical Assistance and Training* (FITT)” (Kershner et al., 2014) addresses threats to implementation fidelity through monitoring of implementation data provided by teachers and observers. In one study, the use of the FITT approach resulted in 98% curricular adherence (Kershner et al., 2014); another study found that more performance feedback resulted in higher implementation (Reinke et al., 2014); and other work supports the utility of social-support networks for teachers confronting similar implementation challenges (Li et al., 2009; Norman & Huerta, 2006). Peer-based mentoring and coaching have also shown promise in improving implementation fidelity of classroom-based interventions (Munthe & Midthassel, 2002; Reinke et al., 2014). Web-based coaching interventions and video guidance appear to improve teachers’ implementation fidelity of EBIs in schools (Schutte et al., 2016; Meyers & Brandt, 2015).

### Implementation of Focus on Youth in the Caribbean (“FOYC”) and Caribbean Informed Parents and Children Together (CImPACT) in the Bahamas

FOYC is an evidence-based, life skills curriculum designed to reduce risk taking behaviors related to HIV/STI transmission and teen pregnancy. CImPACT is a single session intervention including a 24-min educational video filmed in The Bahamas. The video focuses on effective parent-adolescent communication and listening strategies related to difficult topics including “safe-sex” followed by two role-plays for the parent and youth, a discussion, and a condom demonstration. Longitudinal evaluations showed that the intervention significantly increased Bahamian youth’s HIV/AIDS knowledge, perceptions of their ability to use condoms, condom-use intention, and evidence of increased condom use (Chen et al., 2010; Gong et al., 2009).

The Bahamas Ministries of Education and Health in collaboration with our research team implemented FOYC + CImPACT in 66 schools throughout The Bahamas. Since 2018, FOYC + CImPACT was integrated into Health and Family Life Education (HFLE) curriculum in all government grade-6 classes throughout the nation. We use an adapted version of Aaron’s Exploration, Preparation, Implementation, Sustainment (EPIS) model to guide our implementation research. The EPIS model articulates variables that may play crucial roles at different phases in the implementation process (Aarons et al., 2011).

## Study Aims

We conducted a feasibility and optimization study among 81 teachers in 24 schools in New Providence, The Bahamas, to develop and refine several culturally appropriate, theory-driven implementation strategies (e.g., innovative teacher training, implementation monitoring, site-based assistance and mentorship) and to establish the feasibility and preliminary efficacy of implementation strategies. If successful, these teacher training and support strategies will be employed nationally to improve the scale-up of Bahamian school-based prevention programs.

In the present analyses, we sought to address three questions relevant to implementation of an effective HIV prevention program. First, do teachers receiving BMF or SAM show greater implementation fidelity (defied as number of core activities taught) compared to teachers receiving workshop training only? Second, do teachers receiving both BMF and SAM show greater implementation fidelity compared to teachers receiving either BMF or SAM? Third, are teachers’ training experiences, attitudes toward HIV prevention and sex educations in schools, perceptions of principal support, and self-efficacy related to teachers’ implementation performance?

## Methods

### Teacher Training

Eighty-four grade six teachers who teach Health and Family Life Education (HFLE) classes in 24 schools on New Providence completed a two-day teacher training workshop in October 2018. The training was provided by three Bahamian Focus on Youth trainers who have extensive experience implementing FOYC + CImPCT and a US training specialist with expertise preparing educators to lead FOYC + CImPCT. The training focused on increasing the teachers’ curriculum knowledge, building positive attitudes about the curriculum, and increasing skills and comfort to deliver the curriculum. Consistent with the Focus on Youth training guidelines, the training was comprised of clear expressed objectives, short lecturettes, group discussions, videos from the curriculum, skill and curriculum demonstration, active learning through skill practice, role plays and teach backs (Lauer et al., 2014). Additionally, the training aligned with Adult Learning Theory in that the teachers were invited to give input into the training, they participated in several problem solving activities in an environment in which it was safe to make mistakes, and findings from any research in which they were involved could be immediately applied to their work in the classroom (Kearsley, 2010). The teacher training covered: 1) the history and prevalence of HIV and HIV prevention in The Bahamas; 2) overview of FOYC including the research showing its effectiveness; 3) a “walk-through” of each session of FOYC with modeling of the “core” activities (activities considered to be critical to the success of FOYC, such as the family tree activity) and implementation guidance on how to avoid common pitfalls and maximize the impact of each session; 4) a didactic question-and-answer period regarding menstruation, contraception and condom-use; and, 5) a modeling of CImPACT, followed by implementation guidance. All teachers were given a copy of the FOYC teacher training manual. Teachers completed their consent forms, Measures C (Workshop Pre-evaluation), D (Workshop Post-evaluation), and E (Impression before Teaching), at the training workshop. Measures C and D specifically assess teachers’ attendance at the FOYC + CImPACT training workshop and perceptions (before and after training) and past experience about the curriculum. Measure E was administered to the teachers before implementing FOYC + CImPACT to assess factors influencing fidelity of intervention implementation.

In addition to the training workshop, all participating teachers were presented a FOYC + CImPACT 24/7 flash drive for “point-of-care” guidance as they prepared the lessons. FOYC + CImPACT 24/7 is a media-rich digital training program (Firpo-Triplett et al., 2015) accessible anytime, anywhere. Such guaranteed access is important to teachers as the internet connection is sometime unreliable across all of the islands constituting The Bahamas. FOYC + CImPACT 24/7 was based on a similar evidence-based training and implementation support resource (Drake et al., 2015) and provides teachers with information and teaching points about each of the sessions, and intensive modeling and practice designed to develop key skills, such as answering sensitive questions, creating a safe and inclusive classroom, and facilitation skills (Hall & Hord, 2015).

### Teacher Stratification

In preparation for the optimization trial in New Providence in Year 1, the US-Bahamas investigative team identified 81 low-performing and moderate-performing teachers and 10 high-performing teachers using our 7-item pre-implementation school screening tool (Wang et al., 2017). This stratification of teachers into three performance levels was also based on the teachers’ performance during the implementation period (2011–2016) of a prior study using the same curriculum and teachers (Wang et al., 2015) and information regarding their continued implementation of FOYC in their classes over the past year.

In addition, the team held sessions with teachers and administrators from each school to ascertain: their comfort level regarding the curriculum; knowledge and exposure to the FOYC curriculum prior to the 2-day training; and the execution or completion of the FOYC activities post-training (November 2018–January 2019). This information allowed further identification of teachers who were most at-risk for not implementing FOYC + CImPACT.

### School Coordinator and Mentor Training

Twenty-four school coordinators were identified and trained for the purpose of tracking teachers’ implementation and progress biweekly, collecting teacher’s measures, and identifying and reporting issues/problems to the research team in New Providence.

High-performing teachers (mentors) were trained to provide “site-based assistance and mentorship” to at-risk and moderate-performing teachers. Mentors were trained for the purpose of identifying the challenges faced by teachers, assisting teachers in preparing for intervention sessions, and providing guidance to improve curriculum delivery.

The school coordinator and mentor trainings were conducted by two Bahamian trainers who have extensive experience implementing FOYC + CImPACT. Both training sessions lasted 3 to 4 h, respectively.

### Implementation Strategies

The Ministry of Education created a group mentorship program to deploy the strength of high-performing teachers to help teachers who are struggling entitled “Site-based assistance/mentorship” (SAM). High-performing teachers served as team leaders and provided guidance and onsite assistance to low- and moderate-performing teachers to increase the skills and self-efficacy of the latter (Little et al., 2013). SAM is a two-tiered peer mentorship program. *General guidance and biweekly meetings* were established to provide HFLE teachers the opportunity to meet with the team leader biweekly to discuss their progress, identify challenges these teachers are experiencing, and provide guidance during the meeting. *Onsite assistance and observation* were also provided for at-risk or moderate-performing teachers to observe while the session is being taught by a high-performing teacher in the classroom. Teachers who still had difficulties in teaching sensitive topics were observed in the classroom by the team leader who provided onsite assistance. These strategies have evolved as part of The Bahama’s school system’s culture to support new/challenged teachers. An additional program, entitled “biweekly monitoring and feedback (BMF),” was offered to all HFLE teachers to monitor implementation. Teacher implementation was monitored by School Coordinators biweekly with feedback provided to the teachers by the HFLE Senior Curriculum Officer as per MOE policy.

### School-Based Intervention Assignment

Eighty-one at-risk and moderate-performing teachers in 24 schools were randomly assigned to four conditions of the optimization trial in middle January–March 2019, using school-based stratified randomization to avoid possible contamination. More schools were assigned to the control condition or to BFM because only six high-performing teachers/mentors were available for the optimization trial because of their workload/schedule. Nine schools were assigned to the control condition and nine schools to BFM only. Four schools were assigned to SAM only, and two schools were assigned to both BFM and SAM condition. The research protocol was approved by the University of Massachusetts Medical School Human Investigation Committee and the Institutional Review Board of the Bahamian Princess Margaret Hospital, Public Hospitals Authority.

### Measures

#### Implementation Fidelity

To assess implementation, all teachers were asked to complete a Teacher Implementation Checklist specific for each of the eight sessions of FOYC and CImPACT parent session after they had taught the session. The checklist includes the 30 activities identified by the developers as “core elements.” The teachers documented the activities that they covered in each session. Implementation dose was defined as the number of the 30 core activities that were taught during the optimization trial period (middle January to middle March, 2019).

#### Teacher’s Characteristics, Training Experience, and Perceptions

A pre-implementation questionnaire was used to collect information described in the extant research as influencing fidelity of intervention implementation: teacher’s level of formal education; years as a teacher; teacher’s attendance at FOYC training workshop; teachers’ perceptions of the importance of HIV prevention (very important, somewhat important, or not important) for grade six students in their schools; teacher’s comfort level in teaching the FOYC + CImPACT intervention; and teacher’s sense of “ownership” of the curriculum (i.e., perceiving it as a “Bahamian intervention”). In bivariate analyses, responses for years as a teacher were grouped into three collapsed categories (1–10 years, 11–20 years, and > 20 years), and two categories for perceptions of FOYC as a “Bahamian intervention” (very or somewhat/not at all) due to low frequencies in some categories.

The pre-implementation questionnaire assessed teachers’ *autonomy* (four items) (Friedman, 1999), perceived *principal supportiveness* (four items) (Battistich et al., 1997; Liu et al., 2014), teachers’ *confidence* teaching/discussing five topics such as condom use, teen pregnancy, and HIV/AIDS (five items) (Rijsdijk et al., 2014), teachers’ *attitudes toward sex education* in schools (eight items) (Martínez et al., 2014), and teachers’ *self-efficacy* in teaching the FOYC + CImPACT intervention (three items) (Schutte et al., 2014). Answers are given on a Likert scale with five options (1 = totally disagree to 5 = totally agree). The internal consistency of the scales is adequate (Cronbach’s alpha: autonomy, α = 0.79; principal supportiveness α = 0.80; confidence, α = 0.87; attitudes toward sex education, α = 0.77; self-efficacy α = 0.73).

## Analysis

The effect of the implementation strategies (BMF and SAM) on teachers’ implementation was assessed using bivariate and multivariate statistics. ANOVA was used to compare the difference in number of core activities taught by the four groups of teachers. Multiple comparisons were made to examine the difference of all possible pairwise means. The effect of implementation strategies on teachers’ implementation, which was found to be significant at the bivariate level, was further examined using a linear mixed-effects model, controlling for controlling for clustering effect of school and potential confounders, including teachers’ baseline perceptions and number of core activities taught before the start of the optimization trial. In addition, Pearson correlation analysis was conducted to examine the associations among teachers’ perceptions (autonomy, principal supportiveness, attitudes toward sex education in schools, confidence, and self-efficacy) and teachers’ implementation. A parsimonious structural equation model was constructed to examine the relationships among factors influencing teachers’ fidelity of implementation.

## Results

### Association Between Teacher’s Characteristics, Training Experience, Perceptions, and Teacher’s Fidelity of Implementation

Table 1 presents the average number of core activities taught by teachers before or during the optimization trial according to different personal characteristics and training experience. Teachers who completed the FOYC training workshop taught more core activities before the optimization trial (in the fall semester) than did the teachers who did not attend or only attended part of a training workshop (8.36 vs. 4.88, t = 2.19, P < 0.05). Teachers who reported that they had taught several sessions or some activities of FOYC in the past 12 months (prior school year) taught more core activities during the optimization trial period than did the teachers who did not teach any activities of FOYC in the past 12 months (7.57 vs. 7.44 vs. 3.87, F = 6.08, P < 0.01). Teacher’s comfort in teaching FOYC and CImPACT and in leading the roleplays and teachers’ sense of ownership of the FOYC curriculum (e.g., as a “Bahamian intervention”) was positively associated with implementation before the optimization trial. Teacher’s education, years as teacher, and teachers’ perceptions regarding the importance of HIV prevention for grade six youth were not associated with the implementation of FOYC.

**Table 1:** Association between teacher’s characteristics, training experience and number of activities taught before and during the optimization trial among 81 grade six teachers

Variables	No. of teachers^#^	No. of core activities completed before the optimization trial	No. of core activities completed during the optimization trial
Education			
Associate degree/teaching certificate	7	9.29(6.40)	8.00(5.83)
Bachelor’s degree	56	7.11(5.64)	5.88(4.14)
Master’s degree	14	6.50(6.76)	7.43(4.78)
F test		0.54	1.23
Total years as teacher			
1 ~ 10 years	21	5.71(4.26)	6.71(4.65)
11 ~ 20 years	28	7.57(6.83)	6.68(4.55)
> 20 years	32	7.97(5.76)	6.23(4.49)
F test		1.00	0.10
Attended a FOYC training workshop			
Yes	66	7.59(6.04)	6.47(4.33)
No	14	5.57(4.24)	7.29(5.61)
Student’s t test		1.19	0.61
Fully attended training workshop			
Yes	55	8.36(5.99)	6.69(4.53)
No	23	4.88(4.70)	7.47(4.67)
Student’s t test		2.19*	0.62
Taught FOYC in the past 12 months			
No	23	5.78(7.23)	3.87(3.24)
Yes, taught several sessions	44	8.52(5.03)	7.57(4.25)
Yes, only taught several activities	9	5.56(3.54)	7.44(6.09)
F test		2.28	6.08**
Meaningfulness of FOYC for grade 6 youth in your school			
Very meaningful	72	7.57(5.79)	6.33(4.56)
Somewhat meaningful	5	4.60(6.54)	9.20(4.32)
Student’s t test		1.10	1.36
Comfort in teaching FOYC			
Very comfortable	50	8.50(6.39)	6.36(4.35)
Somewhat or not comfortable	27	5.15(3.90)	7.19(5.12)
Student’s t test		2.85**	0.75
Comfort in conducting CImPACT			
Very comfortable	22	9.64(7.36)	5.36(3.39)
Somewhat or not comfortable	52	6.10(4.66)	6.79(4.96)
Student’s t test		2.09*	1.43
Comfort in leading the roleplays			
Very comfortable	47	8.45(6.26)	6.96(4.13)
Somewhat or not comfortable	31	5.61(4.65)	5.77(5.04)
Student’s t test		2.16*	1.13
FOYC is a Bahamian curriculum			
Very	51	8.24(6.34)	7.14(4.72)
Somewhat or not at all	27	5.67(4.25)	5.59(4.29)
Student’s t test		2.13*	1.42

^*^P < 0.05; **P < 0.01. #. 1–7 teachers had missing values in some variables

### Effects of the Implementation Strategies on Teachers’ Degree of Implementation

As shown in Table 2, 81 teachers from 24 schools in New Providence participated in the optimization trial. Results from bivariate analysis indicate that teachers who received both BFM and SAM taught the greatest numbers of core activities, followed by teachers who received either BMF or SAM. Teachers who did not receive BMF or SAM taught the lowest numbers of all activities during the optimization trial period (15.0 vs. 6.9 vs. 7.9 vs. 4.1, p < 0.001). There was no significant difference in number of core activities taught by teachers who received BFM only and teachers who received SAM only.

**Table 2:** Number of core activities taught by teachers during the optimization trial period

Intervention group	Number of schools	Number of teachers	Core activities taught (mean ± SD)	F	P	Paired comparisons
1. Teachers did not receive BFM or SAM	9	31	4.13 ± 3.87	13.20	< .0001	(1, 2) (1,3) (1, 4) (2, 4) (3, 4)
2. Teachers received BFM	9	31	6.94 ± 3.69			
3. Teachers received SAM	4	14	7.93 ± 3.93			
4. Teachers received both BFM and SAM	2	5	15.00 ± 3.54			

BFM = “biweekly monitoring and feedback”; SAM = “site-based assistance and mentorship”

The results of the mixed-effects model indicate that intervention group assignment was significantly related to teachers’ implementation fidelity during the optimization trial (Table 3). Compared to teachers who received both BFM and SAM, teachers who received only one or no intervention demonstrated lower levels of implementation fidelity after controlling for education, training, self-efficacy, attitudes toward sex education, perceived principal support, whether teachers had taught several sessions or some activities of FOYC in the prior school year, number of core activities taught before the start of the optimization trial, and clustering effects of school. Independent of intervention group, teachers’ baseline self-efficacy (P = 0.04) and teachers’ implementation behavior in the prior school year (P = 0.036) were predictive of implementation fidelity during the optimization trial. School random effects were significant, indicating significant variation among schools with regard to teachers’ implementation fidelity (core activities taught). The mixed-effects model was rerun using teachers who did not receive BFM or SAM as the reference group. Teachers who received both BFM and SAM demonstrated higher level of implementation fidelity (β = 8.97, SE = 2.62, t = 3.42, p = 0.0016).

**Table 3:** Mixed-effects model assessing the association between implementation strategies and teachers’ implementation fidelity during the optimization trial

*Fixed effect*	*β*	*SE*	*t*	*p*
Intercept	12.309	4.949	2.49	0.023
Intervention group				
Teachers received none	-8.972	2.624	-3.42	0.002
Teachers received SAM only	-6.950	2.539	-2.74	0.009
Teachers received BFM only	-6.490	2.858	-2.27	0.029
Teachers received both BFM and SAM (ref)	0			
Education				
Associate degree/teaching certificate	0.632	1.848	0.34	0.735
Bachelor’s degree	-0.312	1.119	-0.28	0.782
Master degree (ref)	0			
Taught FOYC in the prior school year	2.233	1.023	2.18	0.036
Number of core activities taught before the trial (first term of current school year)	0.082	0.088	0.92	0.362
Self-efficacy	-1.577	0.742	-2.13	0.041
Attendance at the training workshop	1.067	1.254	0.85	0.401
Attitudes toward sex education	0.518	0.846	0.61	0.544
Perceived principal support	-0.240	0.771	-0.31	0.758
*Random effect*				
School	5.235	3.171	1.65	0.049

### Relationships Among Factors Influencing Teachers’ Fidelity of Implementation

The strength of associations between factors influencing teachers’ self-efficacy and implementation was examined using Pearson correlation coefficients (Table 4). Teachers’ confidence in teaching five core activities, attitudes toward sex education, and perceived principal support were significantly related to increased self-efficacy (r = 0.25–0.48, P < 0.05 or P < 0.001), which in turn was related to teachers’ degree of implementation (r = 0.33, P < 0.01). teachers’ confidence is significantly related to teachers’ positive perceptions of sex education in schools (r = 0.34, p < 0.01). Teachers’ confidence, perceptions of sex education, autonomy and perceived principal support were not significantly related to teachers’ degree of implementation.

**Table 4:** Bivariate correlation among factors influencing teachers’ self-efficacy and implementation before the optimization trial

Variables	1	2	3	4	5	6	7	Mean	SD
1. Confidence	1.00							4.25	0.70
2. Attitudes toward sex education in schools	0.34^**^	1.00						3.57	0.58
3. Autonomy	0.07	-0.01	1.00					3.97	0.64
4. Principal support	0.02	0.02	-0.21	1.00				3.71	0.61
5. Self-efficacy	0.46^***^	0.48^***^	0.01	0.25^*^	1.00			3.60	0.73
6. Attendance at training workshop	0.02	0.22	-0.08	0.02	0.27^*^	1.00		1.78	0.42
7. Number of core activities taught	0.06	-0.05	0.04	0.00	0.33^**^	0.25^*^	1.00	7.15	5.81

^*^p < 0.05; **p < 0.01; ***p < 0.001. SD = Standard deviation. Score range:1 ~ 5 for confidence, sex education, autonomy, principal support and self-efficacy

**Fig. 1:**
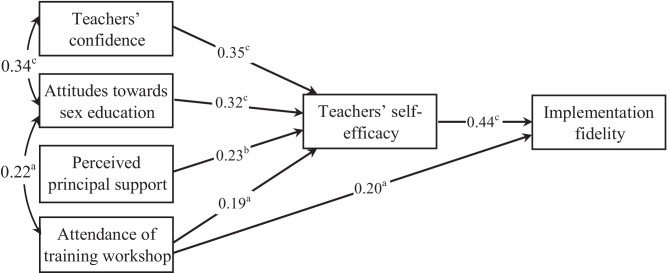
Revised structural model showing relationships among factors influencing teachers’ fidelity of implementation. Standardized path coefficients are shown. Note: a p < 0.05; b p < 0.01; c p < 0.001

Structural equation modeling demonstrated relationships among factors and their direct and indirect effect on fidelity of implementation [i.e., number of core activities taught before the trial (first term of current school year)] (Fig. 1). There were four manifest exogenous variables and two manifest endogenous variables (e.g., self-efficacy, implementation fidelity) in the model. In modifying the initial model, we removed the paths from teachers’ confidence, attitudes toward sex education, and perceived principal support to implementation fidelity as they were nonsignificant. The overall fit of the revised path model was excellent (CFI = 0.99, TLI = 0.93, RMSEA = 0.07, Chi-square = 1.57 p = 0.21; SRMR = 0.02). The analysis revealed an R^2^ value of 0.42 for teachers’ self-efficacy and of 0.21 for fidelity of implementation.

In the revised model, teachers’ confidence, positive attitudes toward sex education in schools, perceived principal support and attendance at the training workshop predicted teachers’ self-efficacy which in turn predicted high-level fidelity of implementation. In addition, teacher’s attendance at the training workshop and their self-efficacy had a direct positive effect on fidelity of implementation. The Sobel test of mediation effect indicated that self-efficacy mediated the relationship between teachers’ confidence, attitudes toward sex education, and perceived principal support and implementation fidelity (z = 2.87, p = 0.004; z = 2.73, p = 0.006; z = 2.41, p = 0.016).

## Discussion

This study developed and refined two theory-driven implementation strategies (BMF and SAM) which demonstrated significant effects on teachers’ fidelity of implementation of an evidence-based HIV intervention in school setting. These strategies, consistent with the principles of community of practice (Li et al., 2009; Norman & Huerta, 2006), have evolved as part of The Bahamian school system’s culture to support teachers who are new in their position and to the program and those who have experienced challenges in implementation. These two implementation strategies developed by our team in collaboration with local teachers and school administrators are culturally appropriate. Our study also shows that teachers’ confidence, attitudes toward sex education, and perceived principal support are significantly related to increased self-efficacy, which in turn is related to teachers’ fidelity of implementation.

Our study identified several pre-implementation factors that are related to teachers’ fidelity of implementation. Although teachers’ attendance at the training workshop was not significantly associated with teachers’ implementation, teachers’ full attendance (completion of the training workshop) was related to increased fidelity of implementation before the optimization trial. Full attendance of the training workshop may reflect teacher’s high motivation and commitment to teaching the intervention curriculum. Teacher training workshops are critical for successful program implementation because they provide the background justification, knowledge, and skills needed to implement the program, increase teachers’ self-efficacy, foster support and commitment to the program, and emphasize the importance of program fidelity (LaChausse et al., 2014; Little et al., 2013). Likewise, teachers’ previous implementation behavior (taught several sessions or some activities of FOYC in the past 12 months) predicted better performance in the following year. Teachers’ sense of community ownership for the intervention was positively related to the fidelity of program implementation before the optimization trial, which is consistent with prior research (Draper et al., 2010). Teachers’ comfort in teaching FOYC and CImPACT and leading roleplays were related to teachers’ fidelity of implementation, which is consistent with findings from previous studies reporting teacher self-efficacy and comfort as significant predictors of implementation (adherence) (LaChausse et al., 2014). Teachers’ comfort level may reflect teachers’ competencies and skills to deliver sensitive topics and to deal with difficulties encountered during the implementation. The absence of an association between teachers’ perceptions regarding the importance of HIV prevention and implementation fidelity may be due to the fact that vast majority of teachers perceived the importance of HIV prevention among youth (94% said it is very meaningful; 6% said it is somewhat meaningful).

Program delivery was a process consisting of several phases, including adoption, implementation, and continuation. Teachers needed support in every phase of the delivery process to enable them to effectively implement the program. Support in the implementation phase is crucial for optimal program effectiveness (Schutte et al., 2016). Our study indicates that pre-implementation teacher training was essential to equip teachers with necessary skills for implementation, but it is not enough. Biweekly implementation monitoring, personal assistance, and mentoring during program delivery were important to ensure teachers’ quality of implementation. Implementation monitoring/assessment acts as a feedback mechanism to improve teachers’ performance and ultimately improve program outcomes (Kershner et al., 2014).

Several lessons were learned from this optimization study. First, selection and training of school coordinators are critically important. School coordinators who are more interested and/or enthusiastic about the program are more likely to encourage and monitor teachers’ implementation. Second, school-based mentorship by experienced teachers provides great support to teachers who experience challenges implementing the curriculum. Mentors need to be identified early so that they can be trained to assist low- or moderate-performing teachers immediately following teachers’ training. Third, transfers and promotions resulted in movement of teachers and school administrators which impacted the sustained implementation of FOYC at the sixth-grade level. There is a need for ongoing training during the school year (especially during summer and teacher professional development periods). Consideration should be given to electronic training (short video support and Webinars series) when face-to-face training is not accessible by some teachers in the more remote family islands.

There are several potential limitations in this study. First, because our study focused on teacher’s implementation behavior and development of two theory-driven implementation strategies to enhance teachers’ implementation, this optimization study did not collect student outcome data, which prevented us from program outcome evaluation. Second, the optimization trial only lasted for two months due to schools’ priority of national examination among grade six students in the second term of school year. Third, our findings were based on teachers’ self-reports of their extent of implementation of the FOYC + CImPACT intervention. It is possible that teachers over-reported their level of implementation. In the current study, trained observers independently observed and assessed approximately 10% of each teacher’s classes. We found that the observer-teacher agreement was high (about 90%), indicating that teachers’ self-reports of their implementation are reliable.

## Conclusion

Our study adds to the sparse but emerging literature on the implementation of evidence-based interventions in school settings. Two theory-driven implementation strategies (BMF and SAM) are effective in promoting teachers’ implementation of youth evidence-based interventions. Our study highlights the importance of selection and training of motivated school coordinators and mentors for successful program implementation. Several lessons learned may be useful for those involved in designing and/or implementing other teacher-delivered school-based health promotion programs. Two theory-driven, effective implementation strategies developed in our study can be used to promote the implementation of effective HIV prevention interventions in schools.

## Data Availability

Data and materials are available for reviewers upon request.

## References

[CR1] Aarons GA, Hurlburt M, Horwitz SM (2011). Advancing a conceptual model of evidence-based practice implementation in public service sectors. Administration and Policy in Mental Health.

[CR2] Battistich, V., Solomon, D., Watson, M., & Schaps, E. (1997). Caring school communities. *Educational Psychologist, 32*(3), 137–151. 10.1207/s15326985ep3203_1

[CR3] Botvin GJ (2004). Advancing prevention science and practice: Challenges, critical issues, and future directions. Prevention Science.

[CR4] Buston K, Wight D, Hart G, Scott S (2002). Implementation of a teacher-delivered sex education programme: Obstacles and facilitating factors. Health Education Research.

[CR5] Chen X, Stanton B, Gomez P, Lunn S, Deveaux L, Brathwaite N, Li X, Marshall S, Cottrell L, Harris C (2010). Effects on condom use of an HIV prevention programme 36 months postintervention: A cluster randomized controlled trial among Bahamian youth. International Journal of STD and AIDS.

[CR6] Collins CB, Sapiano TN (2016). Lessons learned from dissemination of evidence-based interventions for HIV prevention. American Journal of Preventive Medicine.

[CR7] Drake PM, Firpo-Triplett R, Glassman JR, Ong SL, Unti L (2015). A randomized-controlled trial of the effects of online training on implementation fidelity. American Journal of Sexuality Education.

[CR8] Draper CE, Nemutandani SM, Grimsrud AT, Rudolph M, Kolbe-Alexander TL, de Kock L, Lambert EV (2010). Qualitative evaluation of a physical activity-based chronic disease prevention program in a low-income, rural South African setting. Rural and Remote Health.

[CR9] Feldman MB, Silapaswan A, Schaefer N, Schermele D (2014). Is there life after DEBI? Examining health behavior maintenance in the diffusion of effective behavioral interventions initiative. American Journal of Community Psychology.

[CR10] Firpo-Triplett, R., Todd, K., & Rex, P. (2015). FOY 24/7 Online Training. Posted January 2012 (Modified June 2015 FOYC). Monarch Media, Inc. and ETR. http://my.focusonyouth247.com/login/index.php

[CR11] Forman S, Olin S, Hoagwood K, Crowe M, Saka N (2009). Evidence-based interventions in schools: Developers’ views of implementation barriers and facilitators. School Mental Health.

[CR12] Forman SG, Shapiro ES, Codding RS, Gonzales JE, Reddy LA, Rosenfield SA, Sanetti LMH, Stoiber KC (2013). Implementation science and school psychology. School Psychology Quarterly.

[CR13] Friedman IA (1999). Teacher-perceived work autonomy: The concept and its measurement. Educational and Psychological Measurement.

[CR14] Gong J, Stanton B, Lunn S, Deveaux L, Li X, Marshall S, Brathwaite NV, Cottrell L, Harris C, Chen X (2009). Effects through 24 months of an HIV/AIDS prevention intervention program based on protection motivation theory among preadolescents in the Bahamas. Pediatrics.

[CR15] Hall GE, Hord SM (2015). Implementing change: Patterns, principles and potholes.

[CR16] Hill LG, Maucione K, Hood BK (2007). A focused approach to assessing program fidelity. Prevention Science.

[CR17] Kearsley, G. (2010). Andragogy (M. Knowles). *The theory Into practice database*. Retrieved from http://tip.psychology.org. Accessed 5 October 2020.

[CR18] Kershner S, Flynn S, Prince M, Potter SC, Craft L, Alton F (2014). Using data to improve fidelity when implementing evidence-based programs. Journal of Adolescent Health.

[CR19] LaChausse RG, Clark KR, Chapple S (2014). Beyond teacher training: The critical role of professional development in maintaining curriculum fidelity. Journal of Adolescent Health.

[CR20] Lauer P, Christopher D, Firpo-Triplett R, Buchting F (2014). The impact of short-term professional development on participant outcomes: A review of the literature. Professional Development in Education.

[CR21] Li LC, Grimshaw JM, Nielsen C, Judd M, Coyte PC, Graham ID (2009). Evolution of Wenger's concept of community of practice. Implementation Science.

[CR22] Little MA, Sussman S, Sun P, Rohrbach LA (2013). The effects of implementation fidelity towards no drug abuse dissemination trial. Health Education.

[CR23] Liu Y, Ding C, Berkowitz MW, Bier MC (2014). A psychometric evaluation of a revised school climate teacher survey. Canadian Journal of School Psychology.

[CR24] Martínez J, Vicario-Molina I, González E, Ilabaca P (2014). Sex education in Spain: The relevance of teachers’ training and attitudes. Journal for the Study of Education and Development.

[CR25] Meyers, C. V., & Brandt, W. C. (Eds.). (2015). *Implementation Fidelity in Education Research: Designer and Evaluator Considerations*. New York: Routledge, Taylor & Francis Group.

[CR26] Mihalic S, Fagan A, Argamaso S (2008). Implementing the Life Skills Training drug prevention program: Factors related to implementation fidelity. Implementation Science.

[CR27] Molloy LE, Moore JE, Trail J, Van Epps JJ, Hopfer S (2013). Understanding real-world implementation quality and "active ingredients" of PBIS. Prevention Science.

[CR28] Munthe E, Midthassel U (2002). Peer learning groups for teachers: A Norwegian Innovation. New Zealand Annual Review of Education.

[CR29] Norman CD, Huerta T (2006). Knowledge transfer & exchange through social networks: Building foundations for a community of practice within tobacco control. Implementation Science.

[CR30] Proctor, E., Luke, D., Calhoun, A., McMillen, C., Brownson, R., McCrary, S., & Padek, M. (2015). Sustainability of evidence-based healthcare: Research agenda, methodological advances, and infrastructure support. *Implementation Science,**10,* 88. 10.1186/s13012-015-0274-5 10.1186/s13012-015-0274-5 PMC4494699 26062907

[CR31] Reinke, W. M., Stormont, M., Herman, K. C., & Newcomer, L. (2014). Using coaching to support teacher implementation of classroom-based interventions. *Journal of Behavioral Education, 23*(1), 150–167.

[CR32] Renju JR, Andrew B, Medard L, Kishamawe C, Kimaryo M, Changalucha J, Obasi A (2011). Scaling up adolescent sexual and reproductive health interventions through existing government systems? A detailed process evaluation of a school-based intervention in Mwanza region in the northwest of Tanzania. Journal of Adolescent Health.

[CR33] Rijsdijk LE, Bos AE, Lie R, Leerlooijer JN, Eiling E, Atema V, Gebhardt WA, Ruiter RA (2014). Implementation of The World Starts With Me, a comprehensive rights-based sex education programme in Uganda. Health Education Research.

[CR34] Sawyer MG, Harchak TF, Spence SH, Bond L, Graetz B, Kay D, Patton G, Sheffield J (2010). School-based prevention of depression: A 2-year follow-up of a randomized controlled trial of the beyond blue schools research initiative. Journal of Adolescent Health.

[CR35] Schutte, L., Meertens, R. M., Mevissen, F. E., Schaalma, H., Meijer, S., & Kok, G. (2014). Long Live Love. The implementation of a school-based sex-education program in The Netherlands. *Health Education Research,* *29*(4), 583–597. 10.1093/her/cyu021 PMC4101186 24817522

[CR36] Schutte L, van den Borne M, Kok G, Meijer S, Mevissen FE (2016). Innovatively supporting teachers' implementation of school-based sex education: Developing a web-based coaching intervention from problem to solution. Journal of Medical Internet Research.

[CR37] Wang B, Stanton B, Deveaux L, Poitier M, Lunn S, Koci V, Adderley R, Kaljee L, Marshall S, Li X, Rolle G (2015). Factors influencing implementation dose and fidelity thereof and related student outcomes of an evidence-based national HIV prevention program. Implementation Science.

[CR38] Wang B, Stanton B, Lunn S, Patel P, Koci V, Deveaux L (2017). Development of a brief pre-implementation screening tool to identify teachers who are at risk for not implementing intervention curriculum and high-implementing teachers. Health Education & Behavior.

